# Association between adverse childhood experiences and premenstrual disorders: a cross-sectional analysis of 11,973 women

**DOI:** 10.1186/s12916-022-02275-7

**Published:** 2022-02-21

**Authors:** Qian Yang, Edda Björk Þórðardóttir, Arna Hauksdóttir, Thor Aspelund, Jóhanna Jakobsdóttir, Thorhildur Halldorsdottir, Gunnar Tomasson, Harpa Rúnarsdóttir, Hilda Björk Danielsdottir, Elizabeth R. Bertone-Johnson, Arvid Sjölander, Fang Fang, Donghao Lu, Unnur Anna Valdimarsdóttir

**Affiliations:** 1 Department of Medical Epidemiology and Biostatistics, Karolinska Institutet, SE-171 77 Stockholm, Sweden; 2 Institute of Environmental Medicine, Karolinska Institutet, SE-171 77 Stockholm, Sweden; 3 Center of Public Health Sciences, Faculty of Medicine, University of Iceland, IS-101 Reykjavík, Iceland; 4 Department of Psychology, Reykjavik University, IS-101, Reykjavik, Iceland; 5 Department of Biostatistics and Epidemiology, School of Public Health and Health Sciences, University of Massachusetts Amherst, Amherst, MA-01003 USA; 6 Department of Health Promotion and Policy, School of Public Health and Health Sciences, University of Massachusetts Amherst, Amherst, MA-01003 USA; 7 Department of Epidemiology, Harvard TH Chan School of Public Health, Boston, MA-02115 USA

**Keywords:** Adverse childhood experiences, Childhood maltreatment, Household dysfunction, Violence, Premenstrual disorders, PMS, PMDD

## Abstract

**Background:**

Childhood abuse and neglect have been associated with premenstrual disorders (PMDs), including premenstrual syndrome (PMS) and premenstrual dysphoric disorder (PMDD). However, the associations of other adverse childhood experiences (ACEs) and the cumulative number of ACEs with PMDs remain to be explored.

**Methods:**

To evaluate the associations of the cumulative number and types of ACEs with PMDs, we conducted a cross-sectional analysis with a subsample of menstruating women within the Stress-And-Gene-Analysis (SAGA) cohort, assessed for PMDs and ACEs (*N*=11,973). The cumulative and individual exposure of 13 types of ACEs was evaluated by a modified ACE-International Questionnaire. A modified version of the Premenstrual Symptom Screening Tool was used to identify probable cases of PMDs, further sub-grouped into PMS and PMDD. Prevalence ratios (PRs) of PMDs in relation to varying ACEs were estimated using Poisson regression.

**Results:**

At a mean age of 34.0 years (standard deviation (SD) 9.1), 3235 (27%) met the criteria of probable PMDs, including 2501 (21%) for PMS and 734 (6%) for PMDD. The number of ACEs was linearly associated with PMDs (fully-adjusted PR 1.12 per ACE, 95% CI 1.11–1.13). Specifically, the PR for PMDs was 2.46 (95% CI 2.21–2.74) for women with 4 or more ACEs compared with women with no ACEs. A stronger association was observed for probable PMDD compared to PMS (*p* for difference <0.001). The associations between ACEs and PMDs were stronger among women without PTSD, anxiety, or depression, and without childhood deprivation and were stronger among women a lower level of social support (*p* for interaction<0.001). All types of ACEs were positively associated with PMDs (PRs ranged from 1.11 to 1.51); the associations of sexual abuse, emotional neglect, family violence, mental illness of a household member, and peer and collective violence were independent of other ACEs.

**Conclusions:**

Our findings suggest that childhood adverse experiences are associated with PMDs in a dose-dependent manner. If confirmed by prospective data, our findings support the importance of early intervention for girls exposed to ACEs to minimize risks of PMDs and other morbidities in adulthood.

**Supplementary Information:**

The online version contains supplementary material available at 10.1186/s12916-022-02275-7.

## Background

Premenstrual disorders (PMDs), encompassing premenstrual syndrome (PMS) and premenstrual dysphoric disorder (PMDD), refer to a wide range of psychological and physical symptoms which cyclically occur 7–10 days before the onset of menstruation [1]. PMDs are associated with psychiatric comorbidities such as depression and anxiety [1], substantially impaired quality of life [2], and negative health outcomes, such as suicidal behavior [3] and hypertention [4]. The prevalence is estimated to be 20–40% for PMS and 5–8% for PMDD among women of reproductive age worldwide [5]. Similar prevalence rates of PMDs have been documented in teenage girls [6, 7]. Our recent study found that about 70% of women with PMDs reported symptom onset in adolescence [8], lending support to an early-life origin of PMDs. However, the vast majority of previous studies have focused on risk factors in adulthood, such as dietary factors (e.g., lack of calcium/vitamin D) [9], obesity [10], and smoking [11]. It is therefore of paramount importance to identify other risk factors, which are commonly experienced in childhood and adolescence, for PMDs.

About 56% of American women are exposed to at least one adverse childhood experience (ACE), and 14% report experiencing 4 or more ACEs [12]. A growing body of evidence suggests that ACEs are associated with numerous negative health outcomes and social behaviors, such as PTSD, depression, and anxiety [13–15]. However, evidence on the association of ACEs and PMDs remains scarce. One case-control study assessed the history of 5 types of ACEs and found a higher prevalence of PMS among those endorsing 2 or more ACEs [16]. Retrospective data suggest a positive association between childhood abuse, particularly physical and emotional abuse, and PMDs [17] as well as childhood neglect and premenstrual symptoms [18]. To our knowledge, no study has addressed the relationship between other types of ACEs or the cumulative number of ACEs and PMDs.

Evidence suggests that neuroendocrine activation in early life may play a role in the development of PMDs after ACEs. For instance, individuals who are exposed to ACEs show dysregulation in the hypothalamic-pituitary-adrenal (HPA) axis in early life, which may lead to higher susceptibility to developing premenstrual symptoms via traumagenic dynamics and emotional dysregulation [17, 19, 20]. Moreover, posttraumatic stress disorder (PTSD) is a key psychopathology after trauma [21], which has also been associated with PMDD independently of the number or type of previous traumatic events among trauma survivors [22]. Additionally, it is well-documented that ACEs are associated with increased risks of depression and anxiety in adulthood [23] and PMDs are often comorbid with depression and anxiety [24]. Therefore, an observed association between ACEs and PMDs may be moderated by psychiatric comorbid conditions, although few studies have to date addressed this issue [17, 18]. Finally, good social support may help mitigate the negative influence of ACEs [25] and may modify the risk of developing PMDs in adulthood. However, few studies have explored the potential moderation on the association between ACEs and PMDs [17].

## Methods

### Study population

The Stress-And-Gene-Analysis (SAGA) cohort is a prospective cohort in Iceland launched in 2018. All women aged 18–69 years and residing in Iceland as of March 2018 (*n*=104,197) were invited to participate, followed by an electronic questionnaire collecting information on trauma history and health response. All questions in the questionnaire were in Icelandic and the response alternatives for all questions included “can’t/don’t want to answer. In total, 31,064 women consented to participate and 26,905 (86.6%) completed the baseline questionnaire. The demographics of the participants were comparable to the Icelandic female population (manuscript). From among 14,625 women reporting menstruation in the past year and under age 60 at the time of responding, we excluded individuals with >3 missing symptom items on the assessment of PMDs (*n*=224) and those who did not complete the assessment of ACEs (*n*=2428), leaving 11,973 individuals for analysis (Additional file 1: Fig. S1).

### Ascertainment of adverse childhood experiences

A modified Icelandic version of the World Health Organization (WHO) ACE-International Questionnaire (ACE-IQ) was used to assess exposure to ACEs before age 18 [26]. The ACE-IQ consists of 39 items on 13 types of ACEs and has been qualitatively tested in six culturally diverse settings [23]. As exposure to war or collective violence is extremely rare in Iceland, ACE-IQ was modified by adding one screening question on collective violence. The exposure to each type of ACE was defined according to the Guidance for Analyzing ACE-IQ (frequency version) provided by the WHO [26]. In accordance to the guidelines, the specific type of ACEs was counted as exposed if the required minimum frequency of events was met (Additional file 1: Table S1). For example, a person was classified as being physically abused if she answered “many times” on at least one of the two questions regarding physical abuse, while an individual was classified as having a history of sexual abuse if she “ever” experienced any of the 4 items enumerated for sexual abuse.

The accumulative number of ACEs was calculated by summing all ACEs (range: 0–13). The accumulative number of ACEs was calculated by summing all ACEs (range: 0–13). The distribution of ACEs score was summarized in Additional file 1: Fig. S2 and then divided into the categories of 0, 1, 2, 3, ≥4 ACEs, as commonly done in previous studies [27]. We also categorized the 13 types of ACEs into 4 categories as suggested by the WHO: (1) abuse: including physical, emotional, and sexual abuse; (2) neglect: emotional and physical neglect; (3) household dysfunction: witness of family violence, parental separation, substance abuse, and incarceration or mental illness of a household member; and (4) other violence: community violence, peer violence/bullying and collective violence/exposure to war.

### Ascertainment of premenstrual disorders

A modified version of the Premenstrual Symptoms Screening Tool (PSST) was provided to all participants to assess PMDs [28]. The modifications included: 1) combining three separate questions on decreased interest in work, home, and social activities into one question and 2) combining five separate questions assessing the interference of symptoms with work efficiency, relationships with coworkers and family, social life activities, and home responsibilities into one question. The modified PSST assess current premenstrual symptoms and includes 12 items on physical and affective premenstrual symptoms, and one item on the impact of symptoms on daily life, work, and relationships. Each item was rated from 0 (absent) to 3 (severe). Missing symptom items (if <3) were imputed with mean imputation (*N* = 140, 0.1%). The psychometric properties of the PSST have proven excellent in this study (Cronbach alpha = 0.93) and others (positive predictive value of 81–94%) [29, 30].

Using an established classification method [28], probable cases of PMDs were classified as (1) at least one of the four core symptoms (mood swings, irritability, anxiety, depression) reported as moderate to severe; (2) at least 4 of 12 symptoms reported as moderate to severe; and (3) the functioning interference reported as moderate or severe. Furthermore, PMDs were sub-grouped into PMS and PMDD. Probable PMDD cases were classified based on DSM-IV criteria: [31] (1) at least one of the four core symptoms rated as severe and (2) functioning impairment reported as severe. A premenstrual symptom score was calculated and converted to *z* score as a secondary outcome.

### Ascertainment of psychiatric symptoms and social support

Current symptoms of posttraumatic stress disorder (PTSD) were assessed with the PTSD Checklist for DSM-5 (PCL-5) [32]. A validated cutoff score of 33 was used as the threshold for a probable diagnosis of PTSD [33]. The 9-item depression module of the Patient Health Questionnaire (PHQ-9) and the 7-item Generalized Anxiety Disorder scale (GAD-7) were used to assess past 2-week symptoms of depression and anxiety. A cutoff score of 10 was used to define probable cases of depression and anxiety, respectively [34, 35]. Social support may reduce the negative impact of ACEs on mental health [25] and PMDs [11]. Current perceived social support was assessed with the 12-item Multidimensional Scale of Perceived Social Support (MSPSS) and the total score was categorized with quartiles [36]. Missing items for PCL-5 (if ≤6), PHQ-9 and GAD-7 (if≤2 each), and MSPSS (if<3) were imputed with mean imputation, separately.

### Covariates

Information on demographics including participants’ age, height and weight, highest educational level, employment status, current monthly income, marital status, and data on risk factors for PMDs including age at menarche, parity, smoking status, and excessive drinking was collected with the questionnaires [1, 8]. Outliers in height and weight (beyond mean±3 SD) were coded to missing. Excessive drinking was measured as 4 or more drinks on any day (one drink corresponds to a single measure of spirits) [37]. Parity was defined as the number of pregnancies that resulted in a live birth. Due to the known correlation between childhood deprivation and ACEs [38], childhood deprivation was assessed with the question, “Was your family’s economic situation ever so bad that you suffered deprivation? This refers to e.g. lack of nutritious food and/or warm clothes and appropriate footwear during the winter months”. The categorization of covariates is summarized in the Additional file 1: Materials.

### Statistical analysis

We first compared demographic characteristics between women with and without PMDs using *t-*test for age at the survey and chi-square test for other covariates shown in Table 1. We estimated prevalence ratios (PRs) and 95% confidence intervals (CIs) of the accumulative number of ACEs by fitting a log-binomial regression via the robust Poisson regression model [39], since, arguably, PRs are easier to interpret and are of greater clinical relevance. We also compared women who endorsed different numbers of ACEs (1, 2, 3, ≥4) to women without ACEs. To visualize the linear relationship between ACEs and PMDs, we plotted standardized risk and 95% CIs of PMDs over the total number of ACEs using the stdReg package in R [40]. All analyses were adjusted for age and childhood deprivation in Model 1. We additionally adjusted for marital status and socioeconomic factors including educational level, employment status, and income in Model 2. In Model 3, we additionally adjusted for risk factors for PMDs [8, 10, 11, 41], including age at menarche, parity, excessive drinking, smoking status, and BMI. In an additional analysis, we restricted the participants to those with complete information on PMDs assessment. For simplicity, we only employed Model 3 for subsequent analyses.

**Table 1:** Characteristics of women with and without premenstrual disorders (PMDs), number (%) or mean±standard deviation

	No PMDs	PMDs
All	8732	3235
Age (mean±SD)*	36.2±9.4	34.0±9.1
Childhood deprivation*		
Never	7172 (82)	2211 (68)
Rarely	795 (9)	402 (12)
Sometimes	532 (6)	403 (12)
Often	224 (3)	213 (7)
Unknown	9 (0)	6 (0)
Educational level*		
Primary	772 (9)	530 (16)
Secondary school	2099 (24)	916 (28)
College or equivalent	3050 (35)	1004 (31)
Postgraduate	2480 (28)	628 (19)
Unknown	331 (4)	157 (5)
Monthly income (USD)*		
≤$2527	2432 (28)	1305 (40)
$2528–$4212	2525 (29)	998 (31)
$4213–$5897	2150 (25)	569 (18)
≥$5898	1357 (16)	264 (8)
Unknown	268 (3)	99 (3)
Employment status*		
Employed	7687 (88)	2628 (81)
Unemployed	1016 (12)	583 (18)
Unknown	29 (0)	24 (1)
Marital status		
Single/divorced/widowed	2044 (23)	803 (25)
Married/in a relationship	6665 (76)	2413 (75)
Unknown	23 (0)	19 (1)
Age at menarche, years*		
<10	114 (1)	80 (2)
10–11	1524 (17)	697 (22)
12–13	4324 (50)	1556 (48)
14–15	2409 (28)	784 (24)
≥16	361 (4)	118 (4)
Parity*		
0	2652 (30)	1148 (35)
1	1425 (16)	542 (17)
2	2316 (27)	762 (24)
3	1717 (20)	549 (17)
4	388 (4)	147 (5)
≥5	62 (1)	35 (1)
Unknown	172 (2)	52 (2)
Excessive drinking*		
No	7079 (81)	2354 (72)
Yes	1629 (19)	869 (27)
Unknown	24 (0)	12 (0)
Smoking*		
Never	5212 (60)	1491 (46)
Ever	2339 (27)	1075 (33)
Current	1115 (13)	639 (20)
Unknown	66 (1)	30 (1)
BMI, kg/m^2^*		
<18.5	103 (1)	48 (1)
18.5 to 24.9	3514 (40)	1063 (33)
25 to 29.9	2609 (30)	925 (29)
≥30	2259 (26)	1083 (33)
Unknown	247 (3)	116 (4)
Depression^a^*		
No	6972 (80)	1156 (36)
Yes	1756 (20)	2077 (64)
Anxiety^b^*		
No	7368 (84)	1580 (49)
Yes	1361 (16)	1654 (51)
PTSD^c^*		
No	7080 (84)	1558 (49)
Yes	1383 (16)	1647 (51)
Current social support^d^*		
Low	1487 (17)	950 (29)
Medium	4425 (51)	1678 (52)
High	2768 (32)	582 (18)
Unknown	52 (1)	25 (1)

*BMI*, body mass index; *PTSD*, posttraumatic stress disorder

*p<0.001

^a^Depressive symptoms were assessed with the 9-item Patient Health Questionnaire (PHQ)-9 with a score of ≥ 10 points as the cutoff for a probable case

^b^Anxiety symptoms were assessed with the 7-item Generalized Anxiety Disorder (GAD-7) with a score of ≥ 10 as the cutoff for a probable case

^c^PTSD was assessed with the Post-Traumatic Stress Disorder Checklist for DSM-5 (PCL-5) with a score of ≥ 33 as the cutoff for a probable case

^d^Current perceived social support was assessed by the Multidimensional Scale of Perceived Social Support (MSPSS) and categorized with quartiles (Q) (low, 1Q; medium, 2Q–3Q; high, 4Q)

To shed light on the relationship between ACEs and different subtypes of PMDs, we calculated PRs for PMS and PMDD, separately, and compared the difference [42].

Premenstrual symptom *z*-score is another proxy of symptom severity, we then estimated regression slopes (βs) and 95% CIs of ACEs using linear regression.

We also conducted analyses for type-specific ACEs. To adjust for the interrelatedness of different types of ACEs [43], we additionally adjusted for the other 12 types of ACEs to examine the independent association between each type of ACEs and PMDs. ACEs are correlated with psychiatric disorders [23] and the prevalence of psychiatric comorbidities is high among women with PMDs [24]. To examine whether ACE-associated risk of PMDs was modified by psychiatric comorbidities, we performed stratification analyses by adding an interaction term between ACEs and PTSD, depression, and anxiety separately in the model, and estimated PRs in the presence and absence of psychiatric comorbidities. Childhood deprivation is clustered with ACEs and is associated with various adverse health outcomes, we therefore also stratified by childhood deprivation. Moreover, to test the potential influence of recall bias and explore whether the association between ACEs and PMDs diminishes with the time from childhood, we estimated the PRs in different age groups. Last, social support is a protective factor for adverse outcomes after ACEs [25] and may modify the risk of PMDs. Few studies have explored the role of social support for the ACEs-PMDs association [17]. Data was prepared and analyzed in RStudio (Version 1.2.5033) with the statistical significance set at the nominal two-sided 5% level.

## Results

Among 11,973 women (mean age of 35.6 and standard deviation of 9.4 years), 3235 (27%) were classified as probable PMDs, including 2501 PMS (21%) and 734 PMDD (6%). Compared to women without PMDs, women with PMDs were more likely to have suffered childhood deprivation, had lower educational levels or income, and be unemployed and single/widowed at the time of responding. Women with PMDs were younger at menarche, obese, had fewer children, more excessive drinking and smoking, and had probable depression, anxiety, and PTSD (Table 1).

A total of 9211 (77%) women reported at least one ACE. The accumulative number of ACEs was positively associated with PMDs in a graded fashion (fully-adjusted PR 1.12, 95% CI 1.11–1.13; Table 2 and Additional file 1: Fig. S2). The proportion of PMDs increased from 20% among women with no ACEs to 70% among those with ≥4 ACEs (Additional file 1: Fig. S2). Specifically, compared to women with no ACE, women who reported one ACE were about 43% more likely to have PMDs (PR 1.43, 95% CI, 1.27–1.61), while the prevalence was more than two times higher among women with ≥4 ACEs (PR 2.46, 95% CI, 2.21–2.74) (Table 2). A complete-case analysis yielded highly similar results (Additional file 1: Table S2).

**Table 2:** Associations of accumulative adverse childhood experiences (ACEs) with premenstrual disorders (PMDs)

	Women, *N*	PMDs, *N* (%)	Model 1 PR (95% CI)^a^	Model 2 PR (95% CI)^b^	Model 3 PR (95% CI) ^c^
Total number of ACEs (per ACE)	11,967	3235 (27)	1.14 (1.13–1.15)	1.13 (1.11–1.14)	1.12 (1.11–1.13)
By number of ACEs					
0	2756	389 (14)	Ref.	Ref.	Ref.
1	2628	551 (21)	1.49 (1.32–1.67)	1.45 (1.29–1.64)	1.43 (1.27–1.61)
2	2003	526 (26)	1.86 (1.65–2.09)	1.79 (1.60–2.02)	1.76 (1.56–1.97)
3	1468	465 (32)	2.21 (1.96–2.49)	2.12 (1.88–2.38)	2.05 (1.82–2.30)
≥4	3112	1304 (42)	2.73 (2.46–3.04)	2.56 (2.30–2.85)	2.46 (2.21–2.74)

*N*, number; *PR*, prevalence ratio; *CI*, confidence interval

^a^The estimates were adjusted for age at the time of the survey and history of childhood deprivation

^b^The estimates were additionally adjusted for educational level, marital status, employment status, and income

^c^The estimates were additionally adjusted for age at menarche, parity, alcohol intake, smoking status, and BMI

We observed a stronger association for PMDD compared to PMS (PR 1.19 for PMDD vs. 1.10 for PMS; *p* for difference <0.001; Table 3). The difference was particularly evident for women who reported ≥4 ACEs (PR 3.44 for PMDD vs. 2.23 for PMS; *p* for difference <0.001). In addition, positive associations were consistently found for the total premenstrual symptom *z*-score (Additional file 1: Table S3).

**Table 3:** Associations of adverse childhood experiences (ACEs) with premenstrual syndrome (PMS) and premenstrual dysphoric disorder (PMDD)

	Women, *N*	PMS		PMDD		*P* for difference
		cases, *N* (%)	PR (95% CI)^a^	cases, *N* (%)	PR (95% CI)^a^	
Total number of ACEs (per ACE)	11,967	2501 (21)	1.10 (1.08–1.12)	734 (6)	1.19 (1.15–1.22)	<0.001
By number of ACEs						
0	2756	319 (12)	Ref.	70 (3)	Ref.	–
1	2628	453 (17)	1.45 (1.27–1.66)	98 (4)	1.37 (1.01–1.85)	0.516
2	2003	429 (21)	1.77 (1.55–2.03)	97 (5)	1.71 (1.26–2.32)	0.706
3	1468	370 (25)	2.03 (1.77–2.33)	95 (6)	2.15 (1.59–2.92)	0.570
≥4	3112	930 (30)	2.23 (1.97-2.53)	374 (12)	3.44 (2.63–4.50)	<0.001

*N*, number; *PR*, prevalence ratio; *CI*, confidence interval

^a^The estimates were adjusted for age at survey, childhood deprivation, educational level, marital status, employment status, income, age at menarche, parity, alcohol intake, smoking status, and BMI

All individual types of ACEs were positively associated with PMDs after adjustment for all covariates (PRs ranged from 1.11 to 1.51; Table 4); the strongest association was observed for emotional neglect. After mutually adjusting for other types of ACEs, the associations attenuated substantially, yet remained significant for sexual abuse, emotional neglect, family violence, mental illness of a household member, and peer and collective violence (PRs ranged from 0.91 to 1.32).

**Table 4:** Association of an individual type of adverse childhood experiences (ACEs) with premenstrual disorders (PMDs)

	Women, *N*	Cases, *N* (%)	PR (95% CI)^a^	PR (95% CI)^b^
Abuse				
Physical				
No	11,427	2992 (26)	Ref.	Ref.
Yes	540	243 (45)	1.28 (1.16–1.42)	0.95 (0.85–1.06)
Emotional				
No	10,095	2440 (24)	Ref.	Ref.
Yes	1872	795 (42)	1.44 (1.34–1.54)	1.07 (0.99–1.17)
Sexual				
No	8725	2056 (24)	Ref.	Ref.
Yes	3242	1179 (36)	1.37 (1.29–1.46)	1.23 (1.16–1.31)
Neglect				
Physical				
No	11,051	2849 (26)	Ref.	Ref.
Yes	916	386 (42)	1.22 (1.12–1.34)	0.96 (0.88–1.06)
Emotional				
No	8609	1936 (22)	Ref.	Ref.
Yes	3358	1299 (39)	1.51 (1.42–1.61)	1.29 (1.21–1.39)
Household dysfunction				
Family violence				
No	9150	2154 (24)	Ref.	Ref.
Yes	2817	1081 (38)	1.38 (1.29–1.48)	1.10 (1.02–1.19)
Parental separation or divorce				
No	7530	1795 (24)	Ref.	Ref.
Yes	4437	1440 (32)	1.16 (1.09–1.23)	1.05 (0.99–1.12)
Substance abuse				
No	8172	1965 (24)	Ref.	Ref.
Yes	3795	1270 (33)	1.20 (1.13–1.28)	1.01 (0.95–1.08)
Incarcerated household member				
No	11,292	2971 (26)	Ref.	Ref.
Yes	675	264 (39)	1.11 (1.00–1.22)	0.91 (0.82–1.01)
Mental illness				
No	7822	1658 (21)	Ref.	Ref.
Yes	4145	1577 (38)	1.50 (1.41–1.60)	1.32 (1.23–1.41)
Violence				
Community violence				
No	11,502	3027 (26)	Ref.	Ref.
Yes	465	208 (45)	1.31 (1.17–1.45)	1.10 (0.99–1.23)
Bullying				
No	9876	2370 (24)	Ref.	Ref.
Yes	2091	865 (41)	1.39 (1.30–1.49)	1.26 (1.18–1.35)
Collective violence				
No	11,475	3017 (26)	Ref.	Ref.
Yes	492	218 (44)	1.42 (1.29–1.58)	1.25 (1.13–1.38)

*N*, number; *PR*, prevalence ratio; *CI*, confidence interval

^a^The estimates were adjusted for age at survey, childhood deprivation, educational level, marital status, employment status, income, age at menarche, parity, alcohol intake, smoking status, and BMI

^b^The estimates were additionally adjusted for all other ACEs

To test whether the association between ACEs and PMDs was modified by psychiatric comorbidities, we performed stratification analyses and found greater PRs among individuals without probable PTSD, anxiety, or depression (*p* for interaction <0.001; Table 5). Similarly, we performed stratification analysis by social support levels and found greater associations among those with lower social support (*p* for interaction <0.001; Table 5). To alleviate the concern of potential recall bias and to explore the potential modifying role of age on the association between ACEs and PMDs, we conducted stratification analysis and found comparable associations across all groups (Additional file 1: Table S4).

**Table 5:** Associations between accumulative number of adverse childhood experiences (ACEs) and premenstrual disorders (PMDs), stratified by childhood deprivation, psychiatric comorbidities and social support

	Women, *N*	PMDs, *N* (%)	PR (95% CI)^a^
By PTSD			
No	8640	1558 (18)	1.12 (1.10–1.14)
Yes	3034	1647 (54)	1.03 (1.02–1.05)
*P* for interaction			<0.001
By anxiety			
No	8951	1580 (18)	1.14 (1.12–1.16)
Yes	3018	1654 (55)	1.03 (1.02–1.05)
*P* for interaction			<0.001
By depression			
No	8130	1156 (14)	1.12 (1.10–1.14)
Yes	3837	2077 (54)	1.04 (1.03–1.06)
*P* for interaction			<0.001
By childhood deprivation			
Never or rarely	10,585	2613 (25)	1.14 (1.13–1.16)
Sometimes or often	1373	616 (45)	1.05 (1.03–1.07)
*P* for interaction			<0.001
By social support			
Low	3351	582 (17)	1.15 (1.12–1.18)
Medium	6104	1678 (27)	1.11 (1.09–1.12)
High	2441	950 (39)	1.07 (1.06–1.09)
*P* for interaction			<0.001

*N*, number; *PR*, prevalence ratio; *PTSD*, posttraumatic stress disorder

^a^The estimates were adjusted for age at survey, childhood deprivation (except for when stratifying on childhood deprivation), educational level, marital status, employment status, income, age at menarche, parity, alcohol intake, smoking status, and BMI

## Discussion

To the best of our knowledge, this is the first study to comprehensively examine the associations between a cumulative range of ACEs and PMDs in a population-based study. We illustrated a positive, linear relationship between the cumulative number of ACEs and probable PMDs. The association was stronger for the more severe subtype, PMDD, than for PMS. Moreover, the associations were also evident in the absence of common psychiatric comorbidities. All types of ACEs were, albeit to a varying extent, positively associated with PMDs.

Since previous studies on the association between ACEs and PMDs have primarily focused on childhood abuse or neglect [16–18], a comprehensive assessment of the role of different types and numbers of ACEs is needed for an improved understanding of the role of early-life trauma in the development of PMDs. A positive association between exposure to two or more ACEs and PMS was reported recently in a Japanese study [16]. However, the study was based on a clinical sample and the assessment ACEs only included five types. To date, our study is the first to document the associations between 13 common types of ACEs and PMDs in a population-based sample. Although the data on ACEs were collected retrospectively, the consistent findings on the linear and graded relationship between the accumulative number of ACEs and PMDs, as well as the stronger association observed for more severe PMDs (i.e., PMDD vs. PMS) lend support to the role of ACEs in PMDs development later in life.

We found that of all ACEs, emotional neglect was most strongly associated with PMDs, which is in line with previous findings indicating that emotional neglect predicts symptom severity of PMDs [18]. Depression, irritability, and anxiety symptoms are common complaints among individuals exposed to childhood emotional maltreatment [18] and are core symptoms of PMDs [31]. Emotional dysregulation has been reported as an important pathway towards adverse outcomes after childhood adversities [20]. Girls experiencing emotional neglect during childhood/adolescence may develop emotional dysregulation and impaired interpersonal relationships [44], which may exacerbate the impact of PMDs. More importantly, our findings add to the knowledge base that household dysfunction and violence experiences during childhood were also positively associated with PMDs. The associations were particularly strong for mental illness of household members, which might, at least partially, be explained by the genetic liability to psychiatric disorders and PMDs [45, 46]. Our findings on the association between peer-related and collective violence and PMDs highlight the importance of preventing bullying and affirm the devastating effect of collective violence, despite the low prevalence in this specific study population of women.

ACEs are associated with childhood deprivation [47] and childhood deprivation has repeatedly been associated with adverse physical and mental health outcomes in adult life [48, 49]. However, previous studies on early life risk factors for PMDs lack information on childhood deprivation [16–18, 50–52]. Our findings indicated that the association between ACEs and PMDs was independent of (by adjustment) and particularly evident among those without childhood deprivation. These findings not only confirm the previously reported association between ACEs and PMDs, but also reinforce the role of ACEs as important risk factors for PMDs. Social support may help cultivate resilience among individuals exposed to ACEs and thereby mitigate adverse effects on mental health [26]. Indeed, our findings indicated a stronger association between the number of ACEs and PMDs among individuals with a lower level of social support. Future studies are needed to explore the impact of varying sources of social support on this association, such as from family, friends, or community.

ACEs are strongly associated with PTSD [53] and PTSD is associated with subsequent risk of PMDs [54]. Thus, it is possible that PTSD and other symptoms of psychopathology might account for the observed association through a disrupted biological response to stress or emotional dysregulation [22, 55]. Our stratification analysis shows that the observed association between ACEs and PMDs is also present among individuals without psychiatric comorbidities, suggesting that the association cannot be entirely explained by psychiatric comorbidities. The dysregulation of HPA axis after ACEs could lead to higher susceptibility to developing premenstrual syndromes [17, 44]. Larger amygdala volumes after childhood adversities, which is associated with abnormal cortisol response to psychosocial stress, are also noted among women with PMDs [19]. Nevertheless, future research is needed to understand potential biologic underpinnings.

Our study is nested within a large, nationwide-representative cohort of women with detailed, validated measures of ACEs and PMDs. Yet, there are several limitations to be noted. First, recall bias may be introduced when assessing ACEs. However, we observed similar associations across different age bands, presumably, the bias is smaller in younger participants, relieving some concerns that a particular response style or (systematically) biased recall accounts for our findings [56]. Moreover, outcome-dependent misclassification might also be introduced in cross-sectional data, assuming there is a general tendency for women with PMDs to report ACEs. Yet, the observed association in the absence of other psychiatric comorbidities argues against the general tendency assumption. Nevertheless, prospective longitudinal studies are needed to confirm the association between ACEs and PMDs. Third, the probable PMDs were not clinically confirmed. However, given the high sensitivity and relatively low specificity of PSST [29, 30], the misclassification of PMDs would have led to attenuated estimates. The prevalence of PMS and PMDD in our cohort is also comparable to reports from other population-based studies [57, 58]. Last, we cannot exclude the possibility of unmeasured confounding. Risk factors of ACEs, such as low birth weight and birth defects [59], could be another class of covariates. However, it is unknown whether these factors impact on PMD risk, to be able to influence the observed associations.

## Conclusions

Our findings suggest that the cumulative number of ACEs is positively associated with PMDs in adult women, in a dose-response fashion. If confirmed in future prospective studies, these findings motivate refined efforts to prevent ACEs and early intervention for affected girls to reduce the risk of multiple adverse health outcomes, including PMDs.

## Supplementary Information

**Additional file 1:** Supplementary methods, Categorization of covariates. **Figure S1**. Flow chat. **Figure S2**. Distribution of ACE score. **Figure S3**. Standardized risk of PMDs over the total number of ACEs. **Table S1**. Required minimum frequency of events for ascertainment of ACEs. **Table S2**. Associations of accumulative ACEs with PMDs: complete-case analysis. **Table S3**. Associations of ACEs with premenstrual symptom score. **Table S4**. Associations of the number of ACEs with probable cases of PMDs, stratified by age group.

## Data Availability

Data are stored in encrypted form in the data centers of the software company SMART-TRIAL and at the University of Iceland Computing Institute. The procedure was approved by the Icelandic Data Protection Authority. Data cannot be put into a public data repository due to regulations but can be accessed through applications. More information on data application can be found in the following link: https://afallasaga.is/.

## Abbreviations

PMDs: Premenstrual disorders
PMS: Premenstrual syndrome
PMDD: Premenstrual dysphoric disorder
ACEs: Adverse childhood experiences
ACE-IQ: ACE-International Questionnaire
SAGA: Stress-And-Gene-Analysis
PRs: Prevalence ratios
SD: Standard deviation
WHO: World Health Organization
PSST: Premenstrual Symptom Screening Tool
PTSD: Posttraumatic stress disorder
PCL-5: PTSD Checklist for DSM-5
PHQ-9: The 9-item depression module of the Patient Health Questionnaire
GAD-7: The 7-item Generalized Anxiety Disorder scale
MSPSS: Multidimensional Scale of Perceived Social Support
CIs: Confidence intervals
βs: Regression slopes
HPA axis: The hypothalamic-pituitary-adrenal axis
