# Supplementary material for: Association between adverse childhood experiences and premenstrual disorders: a cross-sectional analysis of 11,973 women
Source: BMC Med. 2022 Feb 21;20:60. doi: 10.1186/s12916-022-02275-7 (PMC8859885; doi:10.1186/s12916-022-02275-7)
Supplement: Supplementary file 1 — Additional file 1: Supplementary methods, Categorization of covariates. Figure S1. Flow chat. Figure S2. Distribution of ACE score. Figure S3. Standardized risk of PMDs over the total number of ACEs. Table S1. Required minimum frequency of events for ascertainment of ACEs. Table S2. Associations of accumulative ACEs with PMDs: complete-case analysis. Table S3. Associations of ACEs with premenstrual symptom score. Table S4. Associations of the number of ACEs with probable cases of PMDs, stratified by age group. [file 12916_2022_2275_MOESM1_ESM.docx]

**Association between Adverse Childhood Experiences and Premenstrual Disorders: A Cross Sectional Analysis of 11,973 women**

**Supplementary materials**

Supplementary methods

Categorization of covariates

Highest education level was categorized into: primary, secondary education (high school or vocational education), college or equivalent (BSc or equivalent), and postgraduate (MSc or above). Marital status was divided into: married/in a relationship and single/widowed. Employment status was divided into: employed (including being a student and being on parental leave), and unemployed (including on disability benefits or on sick leave more than 2 months). Monthly income was categorized into: ≤$2527, $2528-$4212, $4213-$5897, and ≥$5898 (conversion rates according to Central Bank of Iceland, October 17, 2018). BMI was categorized into <18.5, 18.5 to 24.9, 25 to 29.9, and ≥30 kg/m^2^. Smoking status was categorized into never smoked, ever smoked or current smoking at the time of responding. Alcohol intake was then categorized into 0, 1-3, and ≥4 drinks. Childhood deprivation was categorized into never, rarely, sometimes and often. Social support was categorized with quantiles (Q): low (Q1), medium (Q2-Q3) and high(Q4).

Figure S1. Flow chart

26,905 participants completed the questionnaires

12,280 excluded:

- 26 aged >60 years
- 12,254 not menstruating during the last year

(among which 8,209 had menopause)

14,625 participants

224 excluded:

>3 PMD symptom items not answered

14,401 participants

2,428 excluded:

did not complete ACEs questionnaire

11,973 participants

Figure S2. Distribution of ACE score.

ACE, adverse childhood experience

Figure S3. Standardized risk of PMDs over the total number of ACEs.

The solid line denotes the standardized risk, while the dash lines indicate the 95% confidence intervals. The estimates were adjusted for age, childhood deprivation, educational level, marital status, employment status, income, age of menarche, parity, alcohol intake, smoking status, and BMI. An interaction term between childhood deprivation and the total number of ACEs was added to improve the prediction.

Table S1. Required minimum frequency of events for ascertainment of ACEs.

| Type of ACEs | Questions | Required minimum frequency |
| --- | --- | --- |
| Abuse | | |
| Physical | Did a parent, guardian or other household member spank, slap, kick, punch or beat you up? | Many times |
|  | Did a parent, guardian or other household member hit or cut you with an object, such as a stick (or cane), bottle, club, knife, whip etc? |  |
| Emotional | Did a parent, guardian or other household member yell, scream or swear at you, insult or humiliate you? | Many times |
|  | Did a parent, guardian or other household member threaten to, or actually, abandon you or throw you out of the house? |  |
| Sexual | Did someone touch or fondle you in a sexual way when you did not want them to? | Ever |
|  | Did someone make you touch their body in a sexual way when you did not want them to? |  |
|  | Did someone attempt oral, anal, or vaginal intercourse with you when you did not want them to? |  |
|  | Did someone actually have oral, anal, or vaginal intercourse with you when you did not want them to? |  |
| Neglect | | |
| Physical | Did your parents/guardians not give you enough food even when they could easily have done so? | Many times |
|  | Were your parents/guardians too drunk or intoxicated by drugs to take care of you? |  |
|  | Did your parents/guardians not send you to school even when it was available? |  |
| Emotional | Did your parents/guardians understand your problems and worries? | Rarely or never |
|  | Did your parents/guardians really know what you were doing with your free time when you were not at school or work? | Rarely or never really |
| Household dysfunction | | |
| Family violence | Did you see or hear a parent or household member in your home being yelled at, screamed at, sworn at, insulted or humiliated? | Many times |
|  | Did you see or hear a parent or household member in your home being slapped, kicked, punched or beaten up? | A few times or many times |
|  | Did you see or hear a parent or household member in your home being hit or cut with an object, such as a stick (or cane), bottle, club, knife, whip etc.? | A few times or many times |
| Parental separation or divorce | Were your parents ever separated or divorced? | Yes |
|  | Did your mother, father or guardian die? |  |
| Substance abuse | Did you live with a household member who was a problem drinker or alcoholic, or misused street or prescription drugs? | Yes |
| Incarcerated household member | Did you live with a household member who was ever sent to jail or prison? | Yes |
| Mental illness | Did you live with a household member who was depressed, mentally ill or suicidal? | Yes |
| Violence | | |
| Community violence | Did you see or hear someone being beaten up in real life? | Many times |
|  | Did you see or hear someone being stabbed or shot in real life? |  |
|  | Did you see or hear someone being threatened with a knife or gun in real life? |  |
| Bullying | How often were you bullied? | Many times |
| Collective violence | During the first 18 years of your life, were you exposed to war/collective violence (e.g. from gangs or police)?* | Yes/No |
|  | Were you forced to go and live in another place due to any of these events? | Ever |
|  | Did you experience the deliberate destruction of your home due to any of these events? |  |
|  | Were you beaten up by soldiers, police, militia, or gangs? |  |
|  | Was a family member or friend killed or beaten up by soldiers, police, militia, or gangs? |  |

*this is a screening question, participants that responded yes recieved four follow-up questions.

Table S2. Associations of accumulative ACEs with PMDs: complete-case analysis.

|  | Women, N | PMDs, N (%) | Model 1  PR (95% CI) ^a^ | Model 2  PR (95% CI) ^b^ | Model 3  PR (95% CI) c |
| --- | --- | --- | --- | --- | --- |
| Total number of ACEs (per ACE) | 11,791 | 3,172 (100) | 1.14 (1.13-1.15) | 1.13 (1.11-1.14) | 1.12 (1.11-1.13) |
| By number of ACEs |  |  |  |  |  |
| 0 | 2,725 | 381 (14) | Ref. | Ref. | Ref. |
| 1 | 2,590 | 544 (21) | 1.50 (1.33-1.69) | 1.47 (1.30-1.65) | 1.45 (1.28-1.63) |
| 2 | 1,976 | 516 (26) | 1.86 (1.66-2.10) | 1.80 (1.60-2.02) | 1.76 (1.56-1.98) |
| 3 | 1,445 | 454 (31) | 2.21 (1.96-2.49) | 2.12 (1.88-2.39) | 2.04 (1.81-2.31) |
| ≥4 | 3,055 | 1,277 (42) | 2.75 (2.47-3.06) | 2.58 (2.31-2.87) | 2.47 (2.22-2.76) |

N, number; PR, prevalence ratio; CI, confidence interval.

^a^ The estimates were adjusted for age at the time of the survey and history of childhood deprivation.

^b^ The estimates were additionally adjusted for educational level, marital status, employment status and income.

^c^ The estimates were additionally adjusted for age at menarche, parity, alcohol intake, smoking status, and BMI.

Table S3. Associations of ACEs with premenstrual symptom score.

| Premenstrual symptoms | Women, N | *zscore*, mean±SD | β (95% CI) ^a^ |
| --- | --- | --- | --- |
| Per ACE | 11,973 | -0.06±0.98 | 0.12 (0.12-0.13) |
| By number of ACEs |  |  |  |
| 0 | 2,756 | -0.46±0.82 | Ref. |
| 1 | 2,628 | -0.23±0.90 | 0.21 (0.16-0.26) |
| 2 | 2,004 | -0.08±0.94 | 0.35 (0.30-0.40) |
| 3 | 1,471 | 0.08±0.96 | 0.48 (0.42-0.54) |
| ≥4 | 3,114 | 0.39±1.03 | 0.76 (0.71-0.81) |

N, number; SD, standard deviation; CI, confidence interval.

^a^ The estimates were adjusted for age, childhood deprivation, educational level, marital status, employment status, income, age of menarche, parity, alcohol intake, smoking status, and BMI.

Table S4. Associations of the number of ACEs with probable cases of PMDs, stratified by age group.

|  | Women, N | Case (%) | PR (95% CI) ^a^ |
| --- | --- | --- | --- |
| By age group, years |  |  |  |
| 18-24 | 2,129 | 730 (34) | 1.10 (1.08-1.12) |
| 25-29 | 1,948 | 550 (28) | 1.12 (1.10-1.15) |
| 30-34 | 1,805 | 499 (28) | 1.12 (1.09-1.14) |
| 35-39 | 2,066 | 596 (29) | 1.11 (1.09-1.14) |
| 40-44 | 1,885 | 480 (25) | 1.12 (1.09-1.15) |
| 45-49 | 1,519 | 287 (19) | 1.16 (1.12-1.19) |
| 50-60 | 621 | 93 (15) | 1.17 (1.10-1.25) |
| P for interaction |  |  | 0.132 |

N, number; PR, prevalence ratio; CI, confidence interval.

^a^ The estimates were adjusted for age, childhood deprivation, educational level, marital status, employment status, income, age of menarche, parity, alcohol intake, smoking status, and BMI.
